# Goutiness

**Published:** 1906-03-03

**Authors:** 


### GOUTINESS.

In a lecture on the symptoms and treatment of goutiness Dr. Leonard Williams [1] urges the use of large draughts of water, coupled with abstention from alcohol, poultry, fish, game, and sweets. Of drugs which possess a general influence in aiding the elimination of the gouty poison, he considers that iodide of potassium stands pre-eminent. "There is no gouty manifestation which does not yield in a large measure to its intelligent employment." The common mistake is a fear of large doses. The commencing dose should never be less than 10 grains, a quantity which is far less likely than smaller ones to cause the coryzal symptoms of iodism. When these latter occur they may be readily dispelled by the conjunction with the iodide of a few minims of Fowler's solution.

Guaiacum also is held to be of great value, and it is recommended that 10 grains of powdered guaiacum should be given with 10 grains of iodide of potash in a cachet. If this quantity of guaiacum causes purging the dose must, of course, be reduced.

Salicylates are of occasional use; the complaint brought against them that they are inert depends in the author's opinion upon the fact that salicylates are commonly prescribed in association with an alkali. "When salicylates are given they should be prescribed either alone or in conjunction with such a drug as nux vomica, which does not influence their chemical medium; for in the body they play the part of acids, and it is in virtue of this part that they do good."

In addition to the above, Dr. Williams believes that both phosphoric and citric acid have their uses in the treatment of gouty manifestations. He holds that citric acid, taken in the form of lemon-juice and in large doses, say, half-an-ounce a day, is a most useful general corrective to the gouty tendency.

[1] Clin. Jour., Jan. 31.

